# Identification of Upregulated Genes under Cold Stress in Cold-Tolerant Chickpea Using the cDNA-AFLP Approach

**DOI:** 10.1371/journal.pone.0052757

**Published:** 2013-01-14

**Authors:** Ali Dinari, Ali Niazi, Ali Reza Afsharifar, Amin Ramezani

**Affiliations:** 1 Institute of Biotechnology, Shiraz University, Shiraz, Iran; 2 Plant Virology Research Center, College of Agriculture, Shiraz University, Shiraz, Iran; 3 Department of Medical Biotechnology, School of Advanced Medical Sciences and Technologies, Shiraz University of Medical Sciences, Shiraz, Iran; Texas A&M University, United States of America

## Abstract

Low temperature injury is one of the most significant causes of crop damage worldwide. Cold acclimatization processes improve the freezing tolerance of plants. To identify genes of potential importance for acclimatzation to the cold and to elucidate the pathways that regulate this process, global transcriptome expression of the chickpea (*Cicer arietinum* L), a species of legume, was analyzed using the cDNA-AFLP technique. In total, we generated 4800 transcript-derived fragments (TDFs) using cDNA-AFLP in conjunction with 256 primer combinations. We only considered those cDNA fragments that seemed to be up-regulated during cold acclimatization. Of these, 102 TDFs with differential expression patterns were excised from gels and re-amplified by PCR. Fifty-four fragments were then cloned and sequenced. BLAST search of the GenBank non-redundant (nr) sequence database demonstrated that 77 percent of the TDFs belonged to known sequences with putative functions related to metabolism (31), transport (10), signal transduction pathways (15) and transcription factors (21). The last group of expressed transcripts showed homology to genes of unknown function (22). To further analyze and validate our cDNA-AFLP experiments, the expression of 9 TDFs during cold acclimatzatiion was confirmed using real time RT-PCR. The results of this research show that cDNA-AFLP is a powerful technique for investigating the expression pattern of chickpea genes under low-temperature stress. Moreover, our findings will help both to elucidate the molecular basis of low-temperature effects on the chickpea genome and to identify those genes that could increase the cold tolerance of the chickpea plant.

## Introduction

In worldwide agriculture, abiotic stress, such as extreme temperature, drought and salinity, is the primary cause of crop damage and reduces average yields for most crop plants by more than 50% [1]. While it is estimated that biotic stress causes a 10 to 20% decrease in crop yields, abiotic stress, by comparison, results in a 50% decrease [2]. In general, morphological, physiological, biochemical and molecular changes, which are the result of both biotic and abiotic stress factors, influence crop growth and development [3]. One of the major factors of abiotic stress is low temperature, which limits the growth productivity and spatial distribution of crop plants worldwide [4]. Cold stress that occurs during late freezes in spring and sudden freezes in autumn preventing plants from realizing their full genetic potential [1], [4], [5].

Cold acclimatization is a process that increases the freezing tolerance in most temperate plants [5]. Exposure to low temperatures during the cold acclimatization process leads to the remodeling of plant cell structures and the reprogramming of gene expression and metabolism [6]. There is evidence indicating that the cell membrane is the primary site that is damaged by freezing due to the formation of ice in the apoplectic space causing a mechanical strain on the cell wall and plasma membrane, thereby leading to cell rupture [7]. On the other hand, the reactive oxygen species (ROS) produced as a result of low temperature stress can attack all basic molecules, such as carbohydrates, lipids, protein and nucleic acids, and therefore result in membrane oxidation [8]. Cold acclimatization can lead to changes in lipid composition, protein, and nucleic acid conformation, as well as the accumulation of carbohydrates. These modifications assist plants in withstanding and repressing the intensive dehydration produced by freezing stress [5], [8]. The key roles of cold acclimatization are to protect and stabilize the integrity of cell membrane rigidification and to prevent disruption by freezing. This ability is vital for plants because cellular membranes have a fundamental role in metabolism.

While one might previously have questioned how plants sense low temperatures, it is now known that plant responses to any environmental signal are mediated by a series of ratios. This mechanism is referred to as signal transduction [4]. Multiple signaling pathways play a role in the response to cold stress, and these signaling pathways may have a significant overlap with each other and with those patterns of gene expression involved in responses to additional stress factors [8]. The perception of cold stress leads to changes in membrane fluidity and increased rigidity, as those membranes have a higher proportion of unsaturated fatty acids [5]. Sensors can perceive the initial stress signal and can then initiate a signaling cascade to translocate the signals through intracellular space; these sensors can also activate nuclear transcription factors to induce the expression of low-temperature-related genes [1].The transcriptome profile is the main level of plant response to environmental stress factors, as temperatures of cold acclimatization cause profound changes in the transcriptome [5]. The basic mechanism of plant responses to abiotic and biotic stresses is the transcriptional control of the expression of stress-related genes.

The chickpea (*Cicer arietinum* L) is an annual plant that is diploid (2n = 2x = 16) and self-pollinating, and it is cultivated around the world. Because of its high nutrient and protein content, it is the third most important legume worldwide. Despite a wide variety of germplasm sources, cultivated chickpea has very little genetic variation [9]. Most knowledge about plant responses to stress concerns model crops such as *Arabidopsis thaliana*, *oryza sativa*, *medicago truncatula*, and *lotus japonicas*; however, information related to the chickpea genome and its response to stress is limited.

In species for which genome information is limited, two methods of investigation include AFLP and cDNA-AFLP; these methods are the best choices for global genome- and transcriptome-level analysis. Using these methods, researchers are able to discover genes on the basis of their polymorphism or differential expression patterns [10]. cDNA-amplified length polymorphism (cDNA-AFLP) is a sensitive, reproducible and efficient technology for the discovery and identification of genes. The main advantages of the cDNA-AFLP method, as opposed to other techniques such as microarrays and gene chips, are that, first, cDNA-AFLP does not require any prior knowledge of gene sequences, and second, its high specificity allows the detection of rarely expressed genes and differentiates among homologous genes, including members of the same gene families. These features make cDNA-AFLP an ideal system for genome-wide expression analysis [11]. The results of various studies have indicated that this technique is able to provide a global vision of gene expression fluctuation induced by a specific stress; the technique is also a useful tool for gene isolation [12].

In this study, the cDNA-AFLP technique was employed to isolate transcripts during low-temperature stress in the chickpea. Furthermore, we demonstrate that the expression of several differentially expressed genes increased during cold acclimatization using real-time RT-PCR. These genes and their possible functions during cold acclimatization are discussed below.

## Methods

### Plant material, growth conditions and cold stress treatment

Chickpea seeds of the Azad variety (a cold-resistant chickpea genotype) were used in this study. The chickpea plants were cultivated and maintained in a greenhouse at an institute of biotechnology (Shiraz University, Iran). Evaluation of germination and growth conditions were conducted, and seven-day-old seedlings were subsequently divided into groups. One group remained in greenhouse conditions as a control, while the other group was transferred to a cold room at 4°C for three days. On the third day of cold-treatment, tissues (leaves and stems) were sampled both from the control- and cold-treated-seedlings, ground with a mortar and pestle and preserved at −80°C.

### RNA extraction and cDNA synthsis

Total RNA was isolated from the frozen tissues of both the cold-treated and control plants utilizing modified RNA extraction buffer containing 100 mM Tris-HCl, pH = 8; 100 mM liCl; 10 mM EDTA, pH = 8; and 1% SDS, according to the hot phenol method described by Verwoevd et al [13]. The RNA quality and integrity was determined by analyzing 4 µl of total RNA by agarose gel electrophoresis; the RNA quantity was also checked using the NanoDrop 1000 spectrophotometer (Wilmington U.S.A). To eliminate any contaminating genomic DNA, samples of RNA were treated, prior to cDNA synthesis, with RNase-free DNase Kit (Fermentas, Hanover, MD). First-strand cDNA was synthesized from 5 µg of total RNA treated with DNase I using 200 U reverse transcriptase (Fermentas). Second-strand cDNA was synthesized using 10 U of DNA polymerase I and RNaseH (Takara Japan) according to the manufacturers' instructions.

### AFLP reactions

Preparation of the cDNA template was performed as described by Bachem et al. [14]. Double-stranded cDNA was first digested with 10 U of *Eco*R1 for 3 h at 65°C followed by digestion with 10 U of *Mse*I for 3 h at 37°C. The digestion products were ligated to *Eco*RI and *Mse*I double-stranded adaptors at 20°C overnight. The adaptors were 5′-CTCGTAGACTGCGTACC-3′ and 3′-CATCTGACGCATGGTTAA-5′ for *Eco*R1 and 5′-GACGATGAGTCCTGAG-3′ and 3′-TACTCAGGACTCAT-5′ for *Mse*I. Ligated cDNA fragments were subsequently amplified by PCR using non-selective *Eco*R1 and *Mse*I primers. The PCR program was as follows: 5 min at 94°C for the initial denaturation and then 30 s at 94°C (denaturation), 45 s at 50°C (annealing), 60 s at 72°C for an extension (30 cycles), followed by 5 min at 72°C. The amplified products were then analyzed on an ethidium bromide-stained 1% agarose gel. The amplification products were diluted 200-fold with sterile water and then used for selective PCR amplification with primers having two selective nucleotides at the 3′ end. The primer combinations were used for selective PCR amplification. Touch-down PCR conditions for selective amplification were as follows: 5 min 94°C for the initial denaturation, followed by 30 s at 94°C, 35 s annealing at 62°C and 1 min at 72°C for an extension. (from 11 cycles, the annealing temperature was reduced in each cycle by 1°C from 62°C to 52°C, and the annealing temperature was then maintained at 52°C for 25 cycles.) The selectively amplified PCR products were then separated on a denaturing 6% polyacrylamide gel (cleaver scientific, England) running at 250 volts for 4 h. The polyacrylamide gels were then silver stained according to Bassam et al. [15], and the cDNA-AFLP fragments were visualized.

### Isolation, cloning and sequence analysis of up-regulated TDFs

On the basis of their presence, absence or up regulated intensity, the bands of interest were selected and cut from the gels with a surgical blade, then dissolved in 50 µl of sterile water with vigorous vortexing for 1 min, and incubated at room temperature for 30 min. One microliter of each dissolved band was re-amplified using the touch-down PCR method and the specific selective nucleotide primers. The re-amplified fragments were then extracted from 1% agarose gels and subsequently cloned into the pTZ vector using the TA Cloning Kit (fermentas) and sequenced (dragon tech). Using the assembly algorithms of Vector NTI Advance 10 software, we produced EST contigs and compared them against the EST sequences in the TIGR (The Institute for Genomic Research) database. As well as the sequencing results were compared against all nucleotide and protein sequences in the GenBank nonredundant database using the BLASTn and BLASTx programs. Finally 48 transcript derived fragments (TDFs) were submitted to GenBank database after six month.

### Real-time PCR

Real-time PCR was performed on RNA obtained from three independent experiments. All samples included equal quantities of RNA. Total RNA was extracted using RNX-Plus buffer (CINNAGEN, Iran). The purified total RNA was quantified with NanoDrop ND 1000 Spectrophotometer (USA). DNase treatment was carried out using the Fermentas DNase Kit (Fermentas, Hanover, MD) according to the manufacturer's instructions. Three micrograms of DNase-treated RNA was used for first strand cDNA synthesis, using 200 U M-Mulv reverse transcriptase (Fermentas) in a 20 µl final volume. Primer design was carried out using Allele ID 7 software for the reference gene and all other genes of interest. The chickpea Elongation factor α (Ef α) gene (AJ004960.1) was used as the reference gene for data normalization (Table 1).

**Table 1:** Sequences of primers used for Real-Time PCR amplification and the resulting product size.

ta	amplicon length (bp)	sequence	primer
52/6	137 bp	ccacggagacccaaactg	copper-f
52/6	137 bp	tgctggaatcaaacggtatc	copper-r
55/6	149 bp	cttcgccttccaacccttcttg	f nuc act
55/6	149 bp	tgctactccattgtttctgaaccc	f nuc act
51/1	154 bp	catagactgtgatgtagc	f phophs
51/1	154 bp	ttcttcttcctcttcctc	r phophs
53/3	105 bp	aaccacacaaacaacaacaac	f t6-451
53/3	105 bp	ctcctcttccttgccttcc	r t6-451
55/8	177 bp	cgtatccgtagcccttgc	f t4-116
55/8	177 bp	ccgccgatgagtcttatcc	r t4-116
52/7	177 bp	gatggttctggctcgtttgg	f hetro
52/7	177 bp	ggtttcccttgatctactgc	r hetro
53/4	171 bp	aggtgaagcgtgtctactatcc	f cystha
53/4	171 bp	caaatgaggcagcaatgtatggg	r cystha
53/3	91 bp	tggggttgattgtgctctg	f alpha 1-4
53/3	91 bp	attcctctgtgctggcttg	r alpha 1-4
50	102 bp	ggataggatataatcaggcagtag	f t11-143
50	102 bp	tcaaacagtaaagtggacagc	r t11-143

Relative Real-time PCR was performed in a total volume of 20 µl containing 1 µl of cDNA, 1× Syber Green buffer and 4 pmol of each primer. The amplification reactions were carried out in a Line Gene K thermal cycler (Bioer, China) under the following conditions: 2 min at 94°C, 40 cycles of 94°C for 10 sec, Ta°C (annealing temperature) for 15 sec and 72°C for 30 sec. After 40 cycles, the amplification products were heated to 95°C to determine their melting curves and confirm their specificity. All amplification reactions were repeated three times under identical conditions and included a negative control and five standard samples. To ensure that the PCR products were generated from cDNA and not genomic DNA, proper control reactions were carried out without the presence of reverse transcriptase. For the Quantitative Real Time PCR data, the relative expression for the genes of interest was calculated using the threshold cycle (CT) method. The CT for each sample was calculated using the Line-Gene K software and the method of Larionov et al. [16]. Accordingly, the amount of expression of target mRNAs over reference values was calculated by the equation 2^−ΔΔCT^ [17], where ΔCT is determined by subtracting the corresponding Ef α CT value (reference gene) from the specific CT of the target (gene of interest) and ΔΔCT is obtained by subtracting the ΔCT of each experimental sample from that of the calibrator sample.

## Results

### Detection and isolation of cold stress–induced TDFs

**Figure 1:**
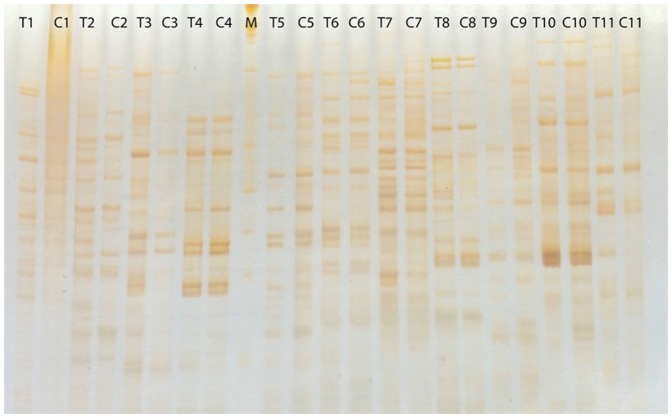
An example of TDFs in a polyacrylamide gel with 13 primer combinations included: EcoR1+AT, CC and Mse1+TG, CT, GC, GA, AG, GT, AT, TT, CG, GG.

In total, 4800 TDFs were generated by 256 primer combinations and visualized on 6% denaturing polyacrylamide gel (Figure 1). The lengths of the TDFs were 50–500 bp in size. The average number of TDFs per lane in the treatment and control samples was the same and was typically 20 TDFs/lane; however, the intensities differed, which implied variation of expression. In total, 102 TDFs with differentially expressed patterns were excised from the gels (as described in methods section) and re-amplified by PCR. Of these, 63 were cloned using the pTZ vector system, and 54 of them were sequenced.

### Gene sequence analysis

Six TDFs were discarded because of vector or adaptor contamination, and the remaining 48 TDFs were compared against the GenBank non-redundant (nr) sequences using the basic local alignment search tool (BLAST). Homology search analysis using the BLASTn and BLASTx programs revealed 22% of TDFs with high homology to ESTs or unknown proteins; 77% of these belonged to known sequences with putative functionality during biotic and abiotic stresses in different plant species. Among those cloned TDFs that had homology with genes of known functions, 31% were involved in metabolism, while 10%, 15% and 21% were involved in transport, signal transduction pathway and transcription factors, respectively One sequence showed no significant homology to any known sequences in public databases. All of these TDF sequences were deposited in NCBI databases (Table 2).

**Table 2:** Transcript derived fragments(TDFs) from chickpea leaves and stems under cold acclimation.

Accession No	Primer com	length	BLAST	score	annotation
Jk649793	GT-TC	375 bp	blastx	2e-57	d-3-phosphoglycerate dehydrogenase
Jk649794	CG-CA	196 bp	blastn	7e-54	protein binding protein (zinc finger family)
Jk649795	AA-GT	233 bp	blastx	2e-23	protein with unknown function
JK649796	AA-GT	184 bp	blastn	3e-72	cystathionine gamma-synthase
Jk649797	AC-CC	328 bp	Blastn	1e-117	zinc finger protein ZF3 gene (*Cicer arietinum*)
JK649798	AC-AG	456 bp	Blastx	5.7	domain binding protein (Laccaria bicolor)
JK649799	AC-TG	304 bp	Blastx	3e-13	heterogeneous nuclear ribonucleoprotein
JK649800	AC-GT	251 bp	Blastx	2e-26	dessication-related protein, putative
JK649801	AG-GG	272 bp	Blastx	3e-35	ptpla domain protein, putative
JK649802	AG-GG	248 bp	Blastx	3e-05	ubiquitin-associated/TS-N domain-containing protein putative
JK649803	AG-TC	223 bp	Blastx	2e-08	mitogen-activated protein kinase 11, putative
JK649804	AC-CT	116 bp	Blastx	0.006	ethylene-responsive element binding factor 4
JK649805	AG-TG	119 bp	Blast est^•^	3e-54	similar to stress related ESTs
JK649806	AT-TC	185 bp	Blastn	4e-36	pyruvate decarboxylase, putative
JK649807	CC-GG	193 bp	Blastn	1e-91	copper amine oxidase
JK649808	CC-CA	153 bp	blastx	5e-15	DNA binding protein
JK649809	CT-CA	260 bp	Blastx	7e-11	inositol or phosphatidylinositol kinase, putative
JK649810	CT-TA	155 bp	Blastx	3e-21	Alpha-1,4-glucan-protein synthase (UDP-forming) cell wall metabolism
JK649811	GA-TC	164 bp	Blastn, blastx	3e-05	unknown function
JK649812	GA-TC	147 bp	Blastn	5e-14	eukaryotic translation initiation factor SUI1, putative
JK649813	GC-TC	121 bp	Blastn	2e-26	C2 domain-containing protein
JK649814	GT-GG	294 bp	Blastn	9e-149	14-3-3-like protein
JK649815	GT-TC	246 bp	Blastx	4e-27	predicted protein
JK649816	GT-TC	256 bp	blastx	9e-22	clathrin assembly protein putative, transport system
JK649817	TA-TC	279 bp	Blastx	5.6	hypothetical protein
JK649818	TC-TC	68 bp	Blast	0.008	Ribulose-1,5 bisphosphate carboxylase/oxygenase large subunit N-methyltransferase, chloroplastic
JK649819	TC-TC	210 bp	Blastn	4e-51	heat shock protein 90
JK649820	TC-TC	151 bp	Blast	1e-09	C3HL domain class transcription factor
JK649821	TC-TC	256 bp	Blastn	7e-95	14-3-3 protein
JK649822	TT-CA	451 bp	blastx	5e-19	Hypothetical protein
JK649823	CG-CC		-		Novel
JK649824	AC-CC	201 bp	Blastx	2e-29	Transport protein particle (TRAPP) component
JK649825	AC-AC	173 bp	Blastx	1e-14	Sodium/calcium exchanger family protein
JK649826	AC-AC	179 bp	Blast	1e-11	MATE efflux protein-related
JK649827	AT-GC	172 bp	Blastx	5e-15	Short-chain dehydrogenase/reductase SDR
JK649828	AT-TA	226 bp	Blastx	2e-07	XH/XS domain-containing protein/XS zinc finger domain-containing protein putative
JK649829	AT-TA	361 bp	Blastx	2e-46	bet1-like snare 1-1(lusin zipper)
JK649830	TG-AG	236 bp	Blastx	6e-10	Chloroplast isoflavone synthase
JK649831	TG-AC	303 bp	Blastx	3e-13	Putative heterogeneous nuclear ribonucleoprotein
JK649832	AC-GC	194 bp	blastx	7e-11	putative glycoside hydrolase family
JK649833	CT-CT	288 bp	Blastx	3e-38	Putative protein COBRA precursor
JK649834	TC-GC	256 bp	blastx	3e-26	clathrin assembly protein, putative
JK649835	CT-TG	440 bp	Blastx	3e-61	Putative AMP dependent ligase
JK649836	GC-AC	186 bp	blastx	2e-06	conserved hypothetical protein
JK649837	GA-TA	298 bp	blastx	9e-35	hydrolase acting on ester bonds
JK649838	CA-CT	258 bp	blastx	7e-11	inositol or phosphatidylinositol kinase
JK649839	TT-CG	142 bp	blastx	2e-13	UDP-D-apiose/UPD-D-xylose synthetase
JK649840	AT-GG	143 bp	Blast est^•^	1e-64	mRNA sequence

### Confirmation of selected gene expression patterns by real-time RT-PCR

**Figure 2:**
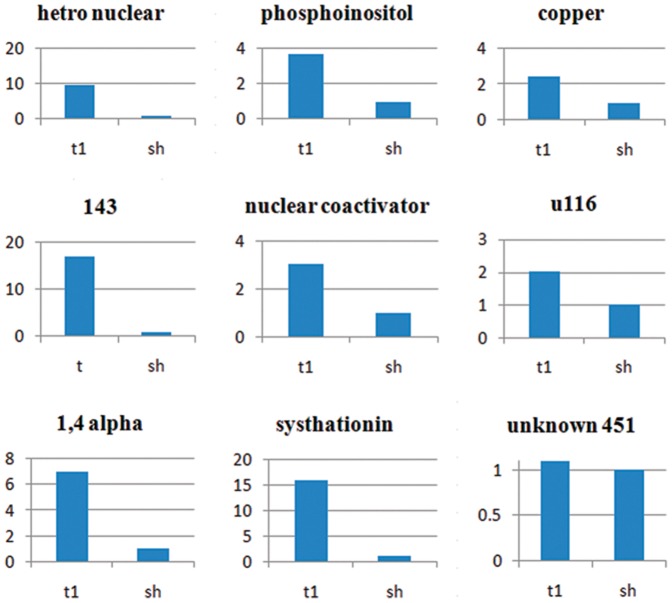
Real-time RT-PCR analysis. Real-time RT-PCR analysis of transcript levels for 9 selected genes in the control- and cold-treated-chickpea leaves. Relative expression for genes of interest were calculated based on the threshold cycle (CT) method. The relative expression level for treated plants at each time point was calculated as fold of the control plants at that time point using the comparative ΔΔCT method. All data were normalized to the Ef α expression level. The mean expression value was calculated for each genes with three replications.

The expression patterns of nine TDFs were analyzed using real-time RT-PCR (Figure 2). The aim was to investigate the reliability of the cDNA-AFLP technique in both detecting differentially expressed genes and validating the levels of gene expression. The TDFs of interest were selected on the basis of their intensity and differential pattern of expression in the cDNA-AFLP experiment. . According to the results we observed that the highest expression is related to cystationine gamma synthase and the TDFs named unknown 143 bp in the treated plants. By the contrary the lower expression is related to unknown 451 bp gene and seems that has similar pattern in the treated and control plant.

## Discussion

The study of plant genomes and the regulation of gene expression under biotic and abiotic stresses demonstrate that any phenomenon of plant life is the result of an interaction between the plant and its environmental conditions. Transcriptome analysis provides a beneficial and strong approach for the investigation of plant-stress interactions [18]. All TDFs discovered in the present study belonged to different categories of genes, including photosynthesis, metabolism, signal transduction, transport systems and other mechanisms related to cold acclimatization. Data obtained from real-time RT-PCR conformed to that of the cDNA-AFLP experiments and confirmed the reliability of our results. Several categories of genes are involved with chickpea compatibility under cold stress conditions. The early transient response to cold stress encompasses genes encoding transcription factors, cell signaling components and those involved in detoxification processes [19]. Whereas the genes active during the late response played a role in metabolism, cell structure and transport systems [2].

Bioinformatics analysis show that 77 percent of TDFs belonged to known genes including genes with putative function related to metabolism (31 percent), transport (10 percent), signal transduction pathways (15 percent) and transcription factors (21 percent). The last group of TDFs show homology to unknown genes. A majorty of TDFs exhibit similarity to genes that response to different environmental stresses. These observation indicate that cross-talk among signal transduction pathways is responsible for different stress conditions [38]. it should be noted that classification of TDFs in above mentioned groups performed using the information of bioinformatics analysis. These finding is according to those of other researches and signified the correctness of our cDNA-AFLP experiments.

### Signal transduction

Approximately 14% of the up-regulated genes in our investigation were involved in signal transduction and plant cell pathways. We noted a 3.5-fold increase in the expression of genes encoding phosphatidylinositol 4–kinase (Jk649809 and Jk649838). Phosphatidylinositol 4–kinase (PI4K) is a key enzyme in phosphatidylinositol metabolism and leads to the release of Ca^2+^ into the cell cytoplasm [1], [20]. The mitogen-activated protein kinase (Jk649803) was up-regulated during cold acclimatization. Genetic studies have demonstrated that the activity and expression of kinases in the MAPK pathway under external stimuli effects such as cold and other stresses are regulated [21]. The formation of ROS is called oxidative stress and acts as a result of environmental stresses [22]. This phenomenon leads to extensive cellular damage and induces genes involved in the detoxification process, such as the copper amine oxidase enzyme gene (JK649807). Our results show that under cold stress, the expression of the copper amine oxidase gene was increased 3-fold and that it may act as a scavenger on these toxic compounds.

### Transcription factors

Numerous studies indicate that transcription factors play pivotal roles in the regulation of temporal and spatial expressions of plant genes responsive to stresses [23]. For example, the Jk649804, Jk649829 and Jk649794 belong to the AP2/EREBP family (ethylene- responsive–element-binding factor or ERF), the bZIP family and the zinc finger family, respectively, and are up-regulated under cold stress conditions. The products of these genes can activate downstream genes through binding to the cis elements (or cis-acting elements) within the promoters of these downstream genes at a later point in time [24]. the gene-encoding heterogeneous nuclear ribonucleoprotein (hnRNP) (Jk649799) was up-regulated during cold stress. Most hnRNP show movement between the nuclear-cytoplasmic space and play a critical role in mRNA export [25], [26]. On the whole, this observation suggests that transcription factors play important roles in the coordinated regulation of cold stress-specific genes.

### Cellular metabolism and organization

Metabolism is not a passive target of cold stress; rather, it can be directly regulated by cold responsive gene expression and cellular organization [27]. The primary role of cold acclimatization is the activation of mechanisms that stabilize membranes and protect cells against the damage occasioned by freezing. Alteration in the lipid composition of plant cell membranes is one among the multiple defense strategies [28]. It is therefore not surprising that the genes involved in fatty acid metabolism were up-regulated by cold stress, including an acyl-lipid desaturases/oleate (Jk649836) and an acetyl-COA synthetase. Genes responsible for numerous cellular activities, including protein modification, degradation enzymes, protein–protein interactions, kinases, and phosphatases, were all up-regulated in our experiments. The genes encoding 14-3-3 protein 9 (JK649814, JK649821) and a heat shock protein (Hsp90) (JK649819) were clear examples in this context. The 14-3-3 protein plays a key role in the activity of various enzymes involved in ion transport and vesicle trafficking and does so via direct protein–protein interactions [29]. Studies suggest that the accumulation of mis-folded and damaged protein due to accumulation of intracellular ROS leads to the activation of Hsp90, which subsequently acts as a molecular chaperone, thereby reducing the harmful effects of ROS in plant cell metabolism [30]. In addition to protein biosynthesis, we observed increases in the expression levels of some genes, such as cystathionine gamma-synthase (JK649796), involved in amino acid biosynthesis. Researchers have demonstrated that this is the primary enzyme active in methionine (met) biosynthesis [31]. An increase in amino-acid content in response to biotic and abiotic stresses as part of plant defense has been reported in several previous studies [32], [33]. Nine of the up-regulated genes encoding proteins function as membrane transporters, such as Na^+^/Ca^2+^ exchanger (JK649825) and clathrin assembly protein (JK649834). The Na+/Ca2+ exchanger is reported to be a member of a large superfamily of membrane proteins that play an important role in Ca^2+^ signaling pathways and that function to transport cytosolic Ca^2+^ across membranes [34]. Clathrin-mediated endocytosis has been reported to be the main mechanism for endocytosis in plant cells [35]. Researchers have demonstrated that the remodeling of the plant cell wall during stress conditions has an essential function in plant resistance. For instance, the gene encoding UDP-D-xylose synthase (JK649839) and the gene encoding COBRA protein (JK649833) proved necessary for cell-wall biogenesis and modification [36], [37].

### Unknown genes

The last group (approximately 22%) of stress up-regulated genes observed in this study is annotated by an unknown sequence. The genes with unknown functions consist of those that have not yet been associated with a response to cold stress, and some of these novel genes did not match any sequence from the GenBank databases. In general, our results confirm previous research, which indicates that multiple defense strategies lead to an enhanced cold temperature defense capacity in plants.

## Supporting Information

List S1 **List of assigned Genbank Accession Numbers.**
(DOCX)
