# Supplementary material for: Identification of Upregulated Genes under Cold Stress in Cold-Tolerant Chickpea Using the cDNA-AFLP Approach
Source: PLoS One. 2013 Jan 14;8(1):e52757. doi: 10.1371/journal.pone.0052757 (PMC3544839; doi:10.1371/journal.pone.0052757)
Supplement: List S1 — List of assigned Genbank Accession Numbers. (DOCX) [file pone.0052757.s001.docx]

The list of assigned Genbank Accession Numbers is below.

dbEST_Id User_Id GenBank_Accn

75843488 eGT-mTC met JK649793

75843489 eCG-mCA fa JK649794

75843490 eAA-mGT pu JK649795

75843491 eAA-mGT se JK649796

75843492 eAC-mCC tr JK649797

75843493 eAC-mAG bi JK649798

75843494 eAC-mTG het JK649799

75843495 eAC-mGT inh JK649800

75843496 eAG-mGG que JK649801

75843497 eAG-mGG sta JK649802

75843498 eAG-mTC JK649803

75843499 eAC-mCT pri JK649804

75843500 eAG-mTG cit JK649805

75843501 eAT-mTC deca JK649806

75843502 eCC-mGG oth JK649807

75843503 eCC-mCA ebd JK649808

75843504 eCT-mCA kin JK649809

75843505 eCT-mTA udb JK649810

75843506 eGA-mTC abm JK649811

75843507 eGA-mTC euk JK649812

75843508 eGC-mTC init JK649813

75843509 eGT-mGG seve JK649814

75843510 eGT-mTC otr JK649815

75843511 eGT-mTC clat JK649816

75843512 eTA-mTC spa JK649817

75843513 eTC-mTC cita JK649818

75843514 eTC-mTC sho JK649819

75843515 eTC-mTC doma JK649820

75843516 eTC-mTC seq JK649821

75843517 eTT-mCA nown JK649822

75843518 mCG-eCC fun JK649823

75843519 mAC-eCC bet4 JK649824

75843520 mAC-eCA chic JK649825

75843521 mAC-eCA pea JK649826

75843522 mAT-eGC prot JK649827

75843523 mAT-eTA aros JK649828

75843524 mAT-eTA shir JK649829

75843525 mTG-eAG amen JK649830

75843526 mTG-eAC leila JK649831

75843527 mAC-eGC behzad JK649832

75843528 mCT-eCT sadjad JK649833

75843529 mTC-eGC faezae JK649834

75843530 mCT-eTG mina JK649835

75843531 mGC-eAC ava JK649836

75843532 mGA-eTA ghorban JK649837

75843533 mCA-eCT dash JK649838

75843534 mTT-eCG sobh JK649839

75843535 mAT-eGG ghorob JK649840
